# Value of diffusion kurtosis MR imaging and conventional diffusion weighed imaging for evaluating response to first-line chemotherapy in unresectable pancreatic cancer

**DOI:** 10.1186/s40644-024-00674-y

**Published:** 2024-02-26

**Authors:** Zehua Zhang, Yuqin Zhang, Feixiang Hu, Tiansong Xie, Wei Liu, Huijing Xiang, Xiangxiang Li, Lei Chen, Zhengrong Zhou

**Affiliations:** 1 Department of Radiology, Minhang Branch, Fudan University Shanghai Cancer Center, No. 106, Ruili Road, 201100 Shanghai, China; 2 Department of Colorectal Surgery, Minhang Branch, Fudan University Shanghai Cancer Center, No. 106, Ruili Road, 201100 Shanghai, China; 3 Department of Radiology, Fudan University Shanghai Cancer Center, No. 270, Dongan Road, 200032 Shanghai, China; 4 Nursing department, Minhang Branch, Fudan University Shanghai Cancer Center, No. 106. Ruili Road, 201100 Shanghai, China

**Keywords:** Diffusion kurtosis imaging, Apparent diffusion coefficient, Unresectable pancreatic cancer, First-line chemotherapy

## Abstract

**Objective:**

To investigate the diagnostic value of diffusion kurtosis magnetic resonance imaging (DKI) and conventional diffusion-weighted imaging (DWI) for evaluating the response to first-line chemotherapy in unresectable pancreatic cancer.

**Materials and methods:**

We retrospectively analyzed 21 patients with clinically and pathologically confirmed unresected pancreatic cancer who received palliative chemotherapy. Three-tesla MRI examinations containing DWI sequences with b values of 0, 100, 700, 1400, and 2100 s/mm^2^ were performed before and after chemotherapy. Parameters included the apparent diffusion coefficient (ADC), mean diffusion coefficient (MD), and mean diffusional kurtosis (MK). The performances of the DWI and DKI parameters in distinguishing the response to chemotherapy were evaluated by the area under the curve (AUC) of the receiver operating characteristic (ROC) curve. Overall survival (OS) was calculated from the date of first treatment to the date of death or the latest follow-up date.

**Results:**

The ADC_change_ and MD_change_ were significantly higher in the responding group (PR group) than in the nonresponding group (non-PR group) (ADC_change_: 0.21 ± 0.05 vs. 0.11 ± 0.09, P *=* 0.02; MD_change_: 0.37 ± 0.24 vs. 0.10 ± 0.12, P *=* 0.002). No statistical significance was shown when comparing ADC_pre_, ADC_post_, MK_pre_, MK_post_, MK_change_, MD_pre_, and MD_post_ between the PR and non-PR groups. The ROC curve analysis indicated that MD_change_ (AUC = 0.898, cutoff value = 0.7143) performed better than ADC_change_ (AUC = 0.806, cutoff value = 0.1369) in predicting the response to chemotherapy.

**Conclusion:**

The ADC_change_ and MD_change_ demonstrated strong potential for evaluating the response to chemotherapy in unresectable pancreatic cancer. The MD_change_ showed higher specificity in the classification of PR and non-PR than the ADC_change_. Other parameters, including ADC_pre_, ADC_post_, MK_pre_, MK_post_, MK_change_, MD_pre_, and MD_post,_ are not suitable for response evaluation. The combined model SUM_change_ demonstrated superior performance compared to the individual DWI and DKI models. Further experiments are needed to evaluate the potential of DWI and DKI parameters in predicting the prognosis of patients with unresectable pancreatic cancer.

## Introduction

With the rapid development of real-time imaging technology, conventional magnetic resonance diffusion techniques have been widely used in pancreatic tissue imaging. The principle of DWI is based on the assumption that the diffusion motion of water molecules follows a normal distribution model in vivo [1]. In addition, the ADC value in the traditional DWI model is also affected by various b values [2].However, diffusion is restricted by the complex microstructure of living tissue and molecular barriers, leading to a non-Gaussian distribution [3, 4]. The DKI model, first proposed by Jensen et al., can be used to identify living tissue using the Gaussian distribution model [5].

As an extension of the diffusion tensor imaging (DTI) model, the DKI model can be used to assess the complexity of microstructures in non-Gaussian tissues [6]. DKI models typically employ high b-values and require at least 3 b-values and a diffusion-sensitive gradient field in 15 directions [7]. DKI uses the same pulse sequence as conventional DWI techniques and tends to adopt larger b values than DWI [8].

DKI technology has shown greater clinical value in tumor detection and staging than traditional DWI technology [1, 9–12].In terms of treatment response evaluation, Granata et al. found that, in locally advanced pancreatic cancer undergoing electrochemotherapy, changes in MD were statistically significant in different efficacy groups (Kruskal Wallis test, *P* = 0.01) [13]. These authors reported believed that the MD_change_ had excellent diagnostic performance for efficacy evaluation (sensitivity = 0.8, specificity = 1.0, AUC = 0.933) [13]. As is known to all, pancreatic cancer is a malignant digestive tumor with poor diagnosis [14]. For those patients who are diagnosed at advanced stage losing their opportunity to undergo radical surgery, first-line chemotherapy regimens recommended by NCCN guidelines are used to prolong the survival period and improve the life of quality [15, 16]. However, few articles have evaluated the efficacy of first-line chemotherapy among unresectable pancreatic cancer patients using DKI. The purpose of our study was to compare the application of DKI technology and traditional DWI technology in evaluating chemotherapy efficacy in patients with unresectable pancreatic cancer.

## Materials and methods

### Clinical data

This retrospective study was approved by Fudan University Shanghai Cancer Center. We enrolled patients with pathologically confirmed PDAC by ultrasound-guided fine-needle aspiration biopsy from August 2021 to December 2022. Thirty-three patients fulfilling the following criteria were included in this study: (1) clinically diagnosed with unresectable pancreatic cancer; (2) without radiotherapy during the treatment phase; (3) three-tesla MRI with a complete DWI sequence before and after chemotherapy could be obtained; (4) the tumor size ≥ 2 cm; and (5) Eastern Cooperative Oncology Group performance status (ECOG PS) score of 0–1. Patients were excluded for incomplete standard treatment (severe adverse reaction, *n* = 3; cytoreductive surgery *n* = 1) or loss to follow-up (*n* = 4). According to these inclusions and exclusions, 21 patients were enrolled in this study. Detailed information about clinical characteristics is listed in Table 1.

**Table 1:** Patient characteristics

Characteristics	Patients
Age(years)	
Mean(range)	64(49–75)
Sex(%)	
Male	71.4(15/21)
Female	28.6(6/21)
Maximum diameter of the lesion(cm)	
Mean (range)	4.7(2.7–7.4)
Lesion location	
Head	52.4(11/21)
Body and tail	47.6(10/21)
Metastasis	
Yes	66.7(14/21)
No	33.3(7/21)
Chemotherapy regimens	
GS	61.9(13/21)
AG	38.1(8/21)

### Chemotherapy regimen

According to the recommendation of comprehensive guidelines for the diagnosis and treatment of pancreatic cancer [16], patients who underwent the following first-line chemotherapy regimens were selected: (1) gemcitabine combined with tegafur gimeracil oteracil potassium (GS): gemcitabine 1000 mg/m^2^ on Day 1 and Day 8, qd, intravenously; tegafur gimeracil oteracil potassium 60 to 100 mg, Day 1–15, bid, orally; every 3 weeks [17]; and (2) gemcitabine combined with nab-paclitaxel (AG): nab-paclitaxel 125 mg/m^2^ and gemcitabine 1000 g/m^2^ on Day 1, Day 8, and Day 15, qd, intravenously, every 4 weeks [18].

### Magnetic resonance examination

Patients underwent scanning using a 3.0-T MR scanner (MAGNETOM Skyra, Siemens Healthcare, Erlangen, Germany) using a 16-channel phased-array volume coil as the receiving coil within 15 days before and after two courses of chemotherapy. MRI sequences included: T1-weighted breath-hold gradient-echo (repetition time (TR), 120 ms; echo time (TE), 1.4 ms; flip angle, 90°; field of view (FOV), 381 × 381 mm^2^; matrix, 320 × 198; number of slices, 42; thickness, 3.5 mm; acquisition time, 32 s) and T2-weighed breath-hold turbo spin‒echo (TR, 3,500 ms; TE, 83 ms; FOV, 381 × 381 mm^2^; matrix, 256 × 256; number of slices, 50; thickness, 4 mm; acquisition time, 3 min 15 s. The conventional DWI were obtained using a free-breathing single-shot echo-planar sequence (TR, 8,500 ms; TE, 56 ms; FOV, 381 × 309 mm^2^; matrix, 256 × 208; number of slices, 28; thickness, 5 mm; b values, 0, 50, 800 s/mm^2^, acquisition time, 2 min 40 s). The DKI sequence used the same scan parameters as the conventional DWI, except the b values (0, 100, 700, 1400, 2100 s/mm^2^), and acquisition time was 4 min 45 s. Big delta time of the mono-polar diffusion gradient is 32.87ms.

### Response evaluation and follow-up

According to the Response Evaluation Criteria in Solid Tumor (RECIST) criteria [19], changes in the primary lesions after 2 cycles of first-line chemotherapy were assessed by an experienced abdominal tumor radiologist. All of the patients were divided into two groups: the PR group (≥ 30% decrease in the sum target lesion diameters, PR group) and the non-PR group (≤ 30% decrease in the sum target lesion diameters, non-PR group). The total number of measurable target lesions was no more than 5 per organ, and repeatable lesions were preferred. The response evaluation criteria are summarized in Table 2.

**Table 2:** Lesion evaluation and overall efficacy evaluation according to the RECIST criteria

Target lesion	Nontarget lesions	New lesions	Overall evaluation
CR	CR	no	CR
CR	Non-CR/Non-PD	no	PR
PR	Non-PD	no	PR
SD	Non-PD	no	SD
PD	/	/	PD
/	PD	/	PD
/	/	yes	PD

Note: CR, complete response; PR, partial response; SD, stable disease; PD, progressive disease

### Measurement of DWI and DKI parameters

ADC values were calculated voxel-wise by fitting the DW images to a mono-exponential signal decay model [20, 21]:

*ADC =* ln(S0/Sb)/b,

in which S_b_ represents the MRI signal intensity with diffusion weighting b, S_0_ represents the intensity without a diffusion gradient, and ADC represents the apparent diffusion intensity.

The DKI parameters were generated voxel-wise by fitting the multi-b DWI to the diffusion kurtosis signal decay equation according to a two-variable linear least squares algorithm [1]:

S(b) = S_0_ × exp (−b·D + 1/6·b^2^D^2^K),

in which the D value represents the corrected diffusion coefficient, and it is different from the conventional ADC value since the D value equals the corrected ADC value in the non-Gaussian model. The K value represents the diffusion kurtosis coefficient, indicating the degree to which the molecular motion deviates from the ideal Gaussian distribution model. Conventional DWI parameters (ADC values) and DKI parameters (MD, MK) were obtained by analyzing multi-b-value DWI parameters using the Body Diffusion Toolbox postprocessing software (Siemens Healthcare GmbH, Erlangen, Germany).

Considering the tumor heterogeneity, the largest area of the tumor was manually selected on the ADC map with reference to the T2-weighted image, as in a previous study [22]. Three regions of interest (ROIs) were placed on the same slice, and the mean ADC, D, and K values were finally calculated, excluding pancreatic ducts, blood vessels, cysts and necrosis. The ROIs were drawn by consensus between two radiologists (with 5 and 10 years of clinical experience in abdominal MR imaging studies). Both radiologists were blinded to the final assessment of chemotherapy response. The corresponding DWI parameters could be obtained by the following equations: ADC_change_ = (ADC_post_ - ADC_pre_) /ADC_pre_, MK_change_ = (MK_pre_ - MK_post_) /MK_pre_; MD_change_ = (MD_post_ - MD_pre_) /MD_pre_; SUM_change_ = ((ADC_post_+MD_post_)-(ADC_pre_+MD_pre_)) / (ADC_pre_+MD_pre_). Among these values, ADC_pre_, MK_pre_, and MD_pre_ represent the values before chemotherapy, while ADC_post_, MK_post_, and MD_post_ represent the values after two courses.

### Statistical analysis

The nonparametric Shapiro-Wilk test and the independent t test were adopted to compare the DWI and DKI parameters among different efficacy groups before and after the treatment. Continuous variables are expressed as the mean ± standard deviation. All statistical analyses were performed using SPSS software (version 22.0, Chicago, IL, USA) and MedCalc software (version 17.5.5, MedCalc Software, Ostend, Belgium). Dependent variables (PR = 1,non-PR = 0) and independent variables (DWI and DKI parameters) were selected for the construction of ROC curves. *P*<0.05 was considered statistically significant. ROC curve analysis was used to evaluated potential variables. Internal validity was assessed by use of bootstrapping procedure. ROC analyses were further performed to evaluate the diagnostic efficacy of each parameter in predicting the chemotherapeutic response of unresectable pancreatic caner.

## Results

### Patient characteristics

**Fig. 1:**
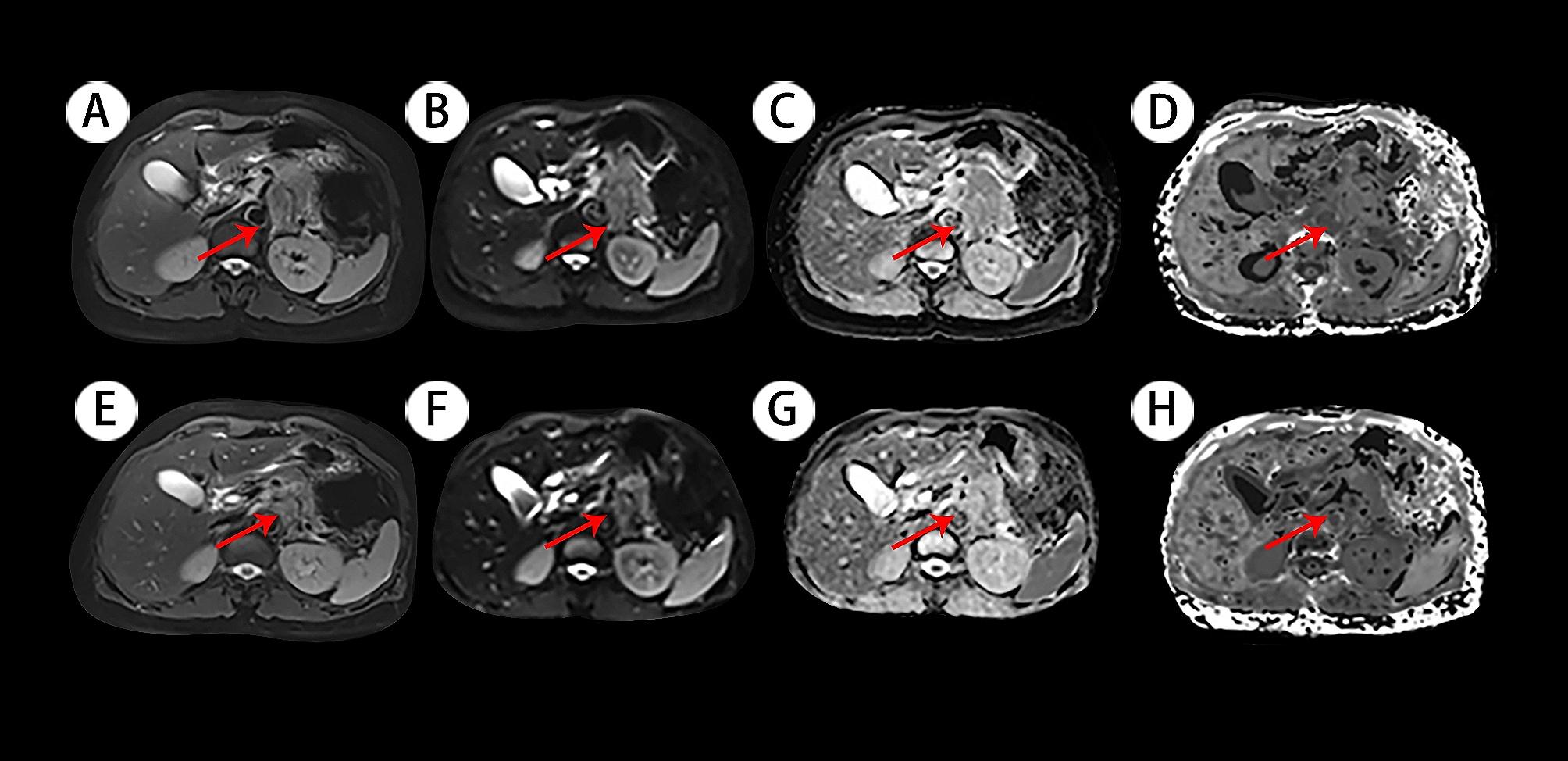
**A** and **E**: Axial half-Fourier-acquired single-shot turbo spin echo (HASTE) T2-weighted images; **B** and **F**: Diffusion weighted images (DWI) with b-value 0 s/mm^2^; **C** and **G**: Apparent diffusion coefficient (ADC) maps; **D** and **H**: Diffusion kurtosis image (DKI) maps. Red arrow: the pancreatic lesion. The images in the first row represent the lesion before undergoing chemotherapy, and the images in the second row represent the lesion that underwent two courses of chemotherapy treatment. Before and after chemotherapy, the ADC and MD values increased significantly, while the MK value decreased slightly. The lesion noticeably shrank after treatment, evaluated as PR according to the RECIST criteria. This patient is a 69-year-old woman with a 4.2 × 3.6 cm mass in the tail of the pancreas and liver metastasis. The following parameters were used: ADC_pre_ (1.06 × 10^− 3^ mm^2^/s), ADC_post_ (1.23 × 10^− 3^ mm^2^/s), ADC_change_ (0.16); MK_pre_ (0.61), MK_post_ (0.60), MK_change_ (-0.01); MD_pre_ (1.61 × 10^− 3^ mm^2^/s), MD_post_ (2.58 × 10^− 3^ mm^2^/s), MD_change_ (0.60)

**Fig. 2:**
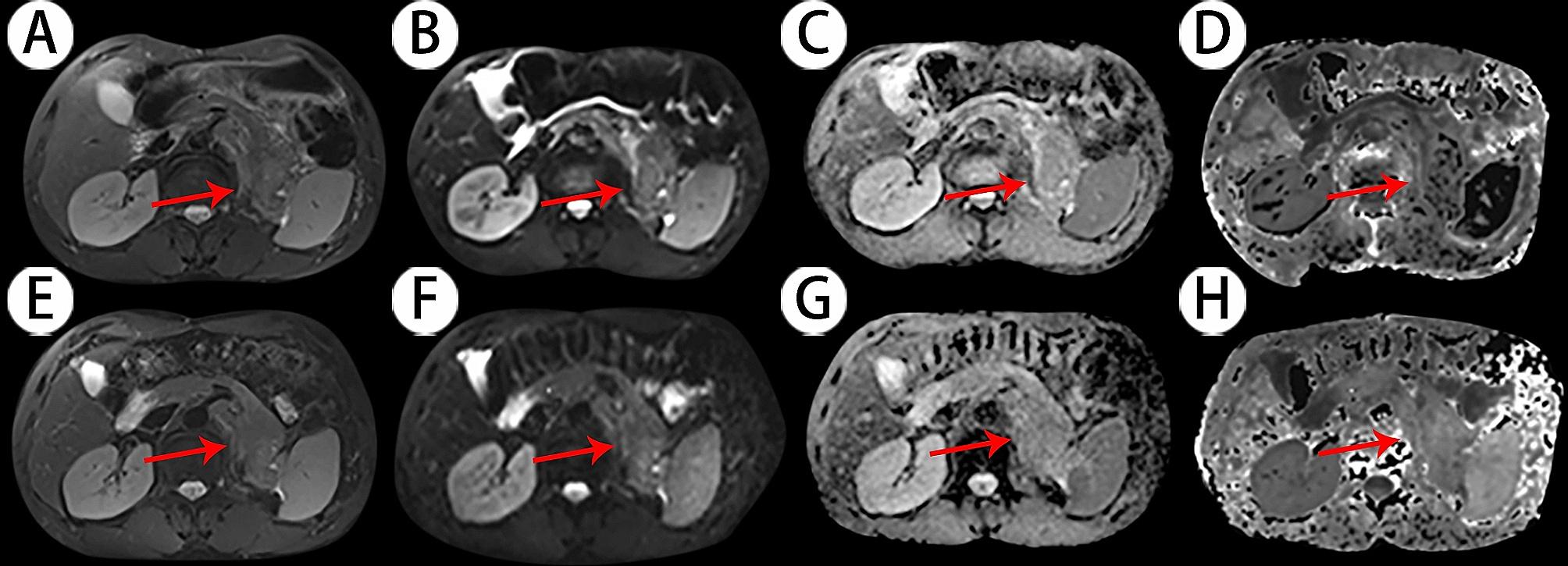
**A** and **E**: T2-weighted images; **B** and **F**: Diffusion weighted image s(DWI) with b-value 0 s/mm^2^; **C** and **G**: Apparent diffusion coefficient (ADC) maps; **D** and **H**: Diffusion kurtosis imaging (DKI) maps. Images **A** to **D** represent the lesion before chemotherapy, while images **E** to **H** represent the lesion after 2 courses of chemotherapy. Red arrow: the pancreatic lesion. The ADC and MD values increased slightly, and the **K** value increased slightly after two courses of chemotherapy. The mass increased slightly after two courses of chemotherapy and was evaluated as non-PR. This patient is a 58-year-old male patient with a 4.8 × 3.5 cm mass in the tail of the pancreas accompanied by liver metastasis. Here are the related parameters: ADC_pre_=1.02 × 10^− 3^ mm^2^/s, ADC_post_=1.12 × 10^− 3^ mm^2^/s, ADCchange = 0.10; MK_pre_=0.64, MK_post_=0.65, MK_change_=0.01; MD_pre_=1.83 × 10^− 3^ mm^2^/s, MD_post_=2.07 × 10^− 3^ mm^2^/s, MD_change_=0.13

The subjects enrolled numbered 21 patients, including 6 women and 15 men, with an average age of 64 ± 8 years old. Patients were classified into the PR group (*n* = 7, Fig. 1) and the non-PR group according to the treatment response (*n* = 14, Fig. 2).

### Statistics of functional parameters

**Fig. 3:**
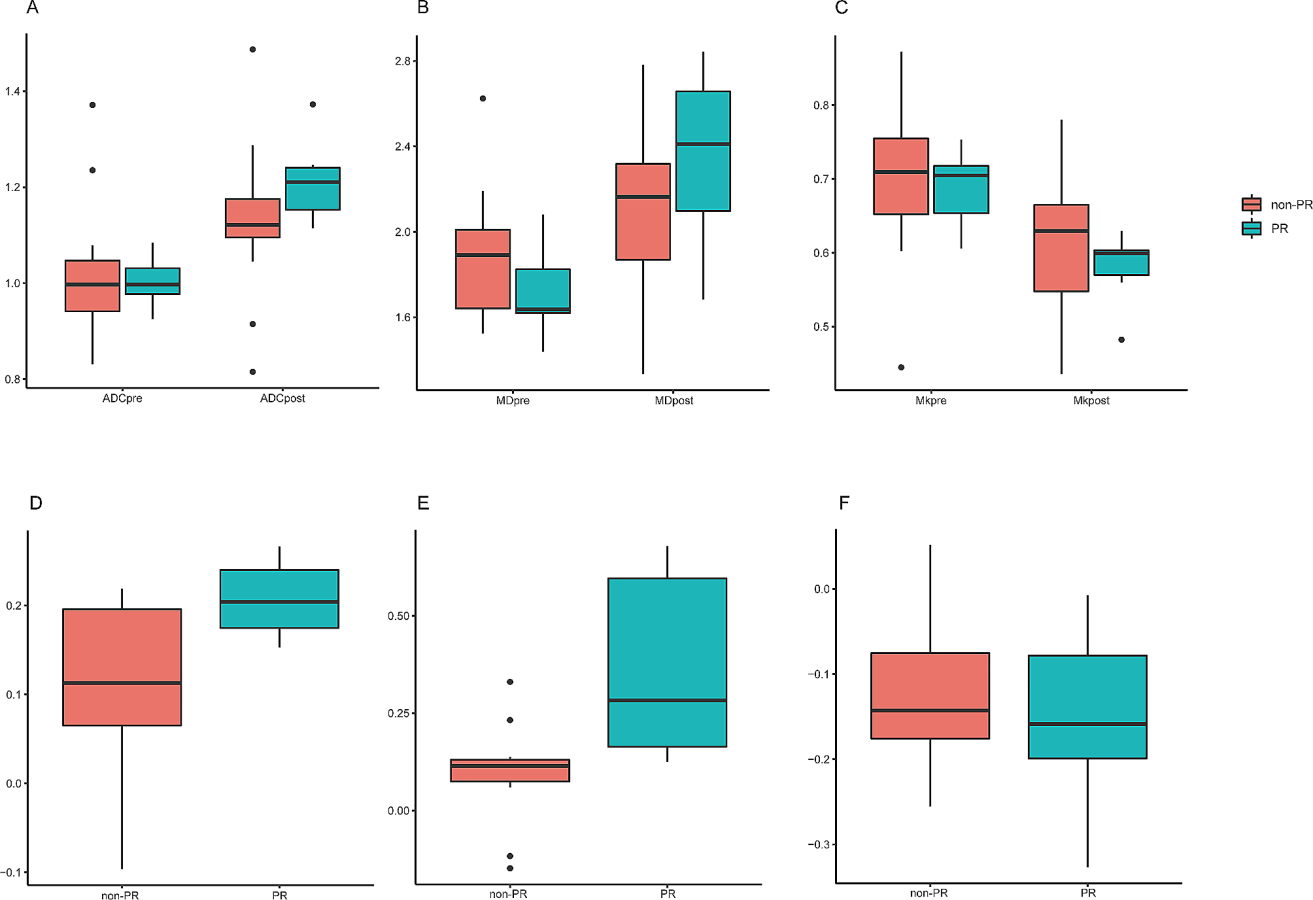
Boxplot of apparent diffusion coefficient (ADC) and diffusion kurtosis imaging (DKI) parameter percentage change values between responders and nonresponders. **A**-**C**:ADC, MD, MK values before and after treatment between PR group and non-PR group. **D**-**F**: ADC_change_, MD_change_, MK_change_ values between PR group and non-PR group

**Fig. 4:**
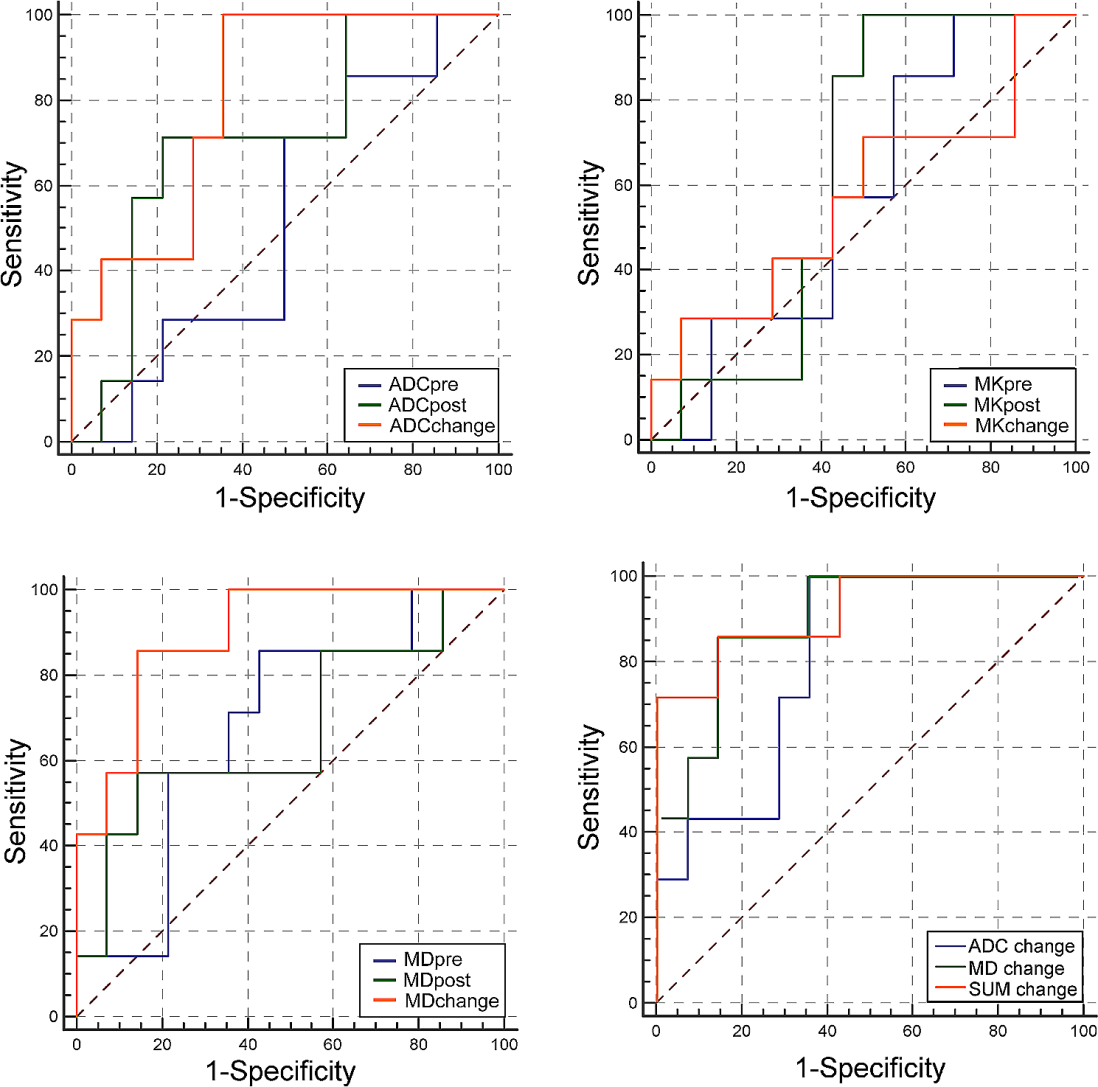
Results of ROC curve analysis evaluating the treatment efficacy using DWI and DKI parameters

The ADC_change_ and MD_change_ in the PR group (0.21 ± 0.05, 0.37 ± 0.24) were significantly higher than those in the non-PR group (0.11 ± 0.09, 0.10 ± 0.12) (*P* = 0.02, 0.002) (Fig. 3). However, no statistically significant differences were shown between the PR group and non-PR group concerning certain aspects of ADC_pre_, ADC_post_, MK_pre_, MK_post_, MK_change_, MD_pre_, and MD_post_ (*P* = 0.734, 0.09, 0.686, 0.289, 0.573, 0.153, 0.166) (Table 3). The ICCs ranged from 0.857 to 0.912 for intraobserver agreement. The AUC of DWI and DKI parameters were successfully validated by 1000 times bootstrapping. The results of ROC curve analysis showed that MD_change_ (AUC = 0.898) had greater diagnostic efficacy than ADC_change_ (AUC = 0.806) (Fig. 4). The MD_change_ had sensitivity of 85.7% and specificity of 85.7%, while the cutoff value was 0.1373. (Tadble 4). The combined model SUM_change_ showed higher AUC (0.912) than that of MD_change_ and ADC_change_ (Fig. 4).

**Table 3:** Differences in ADC value, MK value and MD between the PR group and the non-PR group before and after first-line chemotherapy in patients with unresectable pancreatic cancer

Parameters	PR*	non-PR*	P value
ADC(×10^− 3^mm^2^/s)			
ADC_pre_	1.00 ± 0.55	1.02 ± 0.14	0.734
ADC_post_	1.21 ± 0.09	1.13 ± 0.16	0.09
ADC_change_	0.21 ± 0.05	0.11 ± 0.09	0.02
MK			
MK_pre_	0.67 ± 0.05	0.70 ± 0.11	0.686
Mk_post_	0.58 ± 0.05	0.61 ± 0.09	0.289
Mk_change_	-0.15 ± 0.11	-0.12 ± 0.09	0.573
MD(×10^− 3^mm^2^/s)			
MD_pre_	1.72 ± 0.21	1.89 ± 0.30	0.153
MD_post_	2.35 ± 0.41	2.08 ± 0.40	0.166
MD_change_	0.37 ± 0.24	0.10 ± 0.12	0.002

Note: *All of the data represent the mean ± standard deviation; MK_change=(_MK_pre−_MK_post)/_MK_pre;_ MD_change=(_MD_post−_MD_pre)/_MD_pre;_ ADC_change=(_ADC_post−_ADC_pre)/_ADC_pre_

**Table 4:** ROC curve of DWI and DKI parameters in differentiating chemotherapy efficacy in patients with unresectable pancreatic cancer

Parameters	AUC	95%CI	P value	Sensitivity	Specialty	Cut off value	Youden’s index*
ADC_pre_	0.52	0.295–0.740	0.8798	0.714	0.5	>0.9838	0.2143
MK_pre_	0.571	0.340–0.782	0.5851	1	0.286	≤ 0.7531	0.2857
MD_pre_	0.684	0.447–0.866	0.1484	0.857	0.571	≤ 1.8623	0.4286
ADC_post_	0.714	0.478–0.887	0.0872	0.714	0.786	>0.9838	0.5
MK_post_	0.633	0.397–0.829	0.2935	1	0.5	≤ 0.6295	0.5
MD_post_	0.67	0.437–0.859	0.21	0.57	0.86	>2.3917	0.43
ADC_change_	0.806	0.577–0.944	0.0017	1	0.643	>0.1369	0.6429
MK_change_	0.571	0.340–0.782	0.6295	0.286	0.929	≤-0.2301	0.2143
MD_change_	0.898	0.687–0.986	<0.0001	0.857	0.857	>0.1373	0.7143
SUM_change_	0.912	0.788–0.988	0.002	0.857	0.857	>0.1477	0.7143

*Youden’s index = sensitivity − (1 - specificity); 95% CI, 95% confidence interval;SUM_change_ = ((ADC_post_+MD_post_)-(ADC_pre_+MD_pre_)) / (ADC_pre_+MD_pre_)

## Discussion

Currently, several studies have been reported distinguishing pancreatic ductal adenocarcinoma tissue from surrounding normal pancreatic tissue using DKI [23, 24]. In addition, many scholars have used DKI technology to evaluate the therapeutic effects on tumors in other parts. The purpose of our study was to compare the value of two diffusion techniques, DWI and DKI, in evaluating first-line chemotherapy in unresectable pancreatic cancer.

ADC values are affected by a variety of factors, including intracellular structures, cell membrane integrity, extracellular fiber composition, and more. In patients with unresectable pancreatic cancer, liquefaction, necrosis, fibrosis, loss of cellular structural integrity, and reduction of molecular barriers restricting the diffusion and movement of water molecules eventually lead to changes in corresponding diffusion parameters, providing a theoretical basis for our experimental design. Some scholars have claimed that a low ADC value before treatment often predicts poor prognosis of patients [25]. Conversely, Niwa et al. suggested that lower ADC values before chemotherapy were associated with shorter progression-free survival (PFS) after treatment of patients with resectable pancreatic cancer [26]. However, no significant differences were shown in ADC_pre_ or ADC_post_ between the partial remission group (1.00 ± 0.55 × 10^− 3^ mm^2^/s, 1.21 ± 0.09 × 10^− 3^ mm^2^/s) and the nonpartial remission group (1.02 ± 0.14 × 10^− 3^ mm^2^/s, 1.13 ± 0.16 × 10^− 3^ mm^2^/s) in our study (*P* = 0.734, *P* = 0.09). This result is consistent with the findings of a previous study10. We found that the ADC_change_ in the PR group (0.21 ± 0.05) was significantly higher than that in the non-PR group (0.11 ± 0.09) (*P* = 0.02). Nishiofuku et al. also found that the ADC_ratio_ measured after four weeks of chemotherapy could predict chemotherapy sensitivity and was the most effective independent predictor of PFS (hazard ratio, 4.5; 95% confidence interval, 1.7–11.9; *P* = 0.002), which was not validated in our study [22].

Using the DKI model, we obtained two quantitative parameters, including the kurtosis value (K, indicating the degree of deviation from a Gaussian distribution) and diffusion coefficient (D, defined as the corrected ADC value from a non-Gaussian distribution)1. Compared with ADC, the mean kurtosis coefficient (MK) and mean diffusion coefficient (MD) could provide more information about tissue heterogeneity, vascularity and cellularity [27]. Philipp et al. suggested that the DKI-derived parameter D could be used as a noninvasive marker to assess the interstitial tissue component in pancreatic ductal adenocarcinoma, and it makes benefit for the diagnosis [23]. Among the diffusion parameters, the MD among the DKI parameters has been discovered to distinguish between among pancreatic parenchyma, peri-lesional inflammation and pancreatic tumors [24].

Currently, DKI technology has been widely used to evaluate the treatment efficacy in tumors of other parts of the body. In assessing the effect of induction chemotherapy (IC) on the treatment of locoregionally advanced nasopharyngeal carcinoma, Zhao et al. found that the mean MK (*P* < 0.001) values decreased dramatically, while MD (*P* < 0.001) significantly increased after treatment; additionally, ADC_pre_, MD_pre_ and MK_ratio_ were not significantly different between the two groups [28]. In contrast to their outcomes, in our study, there were no significant differences in MK_pre_ or MK_post_ between the PR group (0.67 ± 0.05, 0.58 ± 0.05) and the non-PR group (0.70 ± 0.11, 0.61 ± 0.09) (*P* = 0.686, *P* = 0.289). Some investigators have indicated that the influence of external factors on the K value is more obvious than that on the D value, such as breathing motion, noise, and artifacts1. Some studies have also shown that a decrease in the D value is not necessarily accompanied by an increase in the K value from normal tissues or tumor tissues [29]. In this trial, there was no significant difference in the alteration of the K value between the PR group and the non-PR group before and after chemotherapy. The reasons for the differences in the above conclusions could include: (1) different ROI segmentation; (2) various selections of multiple b values; 3)the diffusion time used in the single-shot EPI sequence; and 4) a variety of efficacy evaluation standards and postprocessing equipment and processing schemes.

Tumor tissue liquefaction necrosis and fibrosis were more obvious in the PR group than in the non-PR group, and the histological changes could be reflected by the ADC_change_ and MD_change_. The MD_change_ in the PR group (0.37 ± 0.24) was significantly higher than that in the non-PR group (0.10 ± 0.12) (*P* = 0.002). The results of our study are consistent with those of Wu Rui etc [30], who claimed that the MD_ratio_ was significantly different between the PR group and the non-PR group in patients with cervical (neck) non-Hodgkin lymphoma (NHL), showing a significant, positive correlation and high agreement with the ADC_ratio_ (*r* = 0.776, *P* < 0.001). Similarly, we found that MD_change_ had a larger AUC (0.898 vs. 0.806) and higher specificity (0.857 vs. 0.643) than ADC_change_, which can be explained by the difference between the DKI model and DWI model since MD should better reflect the motion state of free water. In addition, the SUM_change_ showed the highest AUC (0.912) as a combined model of DWI and DKI parameters, which indicated that the combination of DWI and DKI technique may contribute to further supplementation.

Our study has the following shortcomings. First, the sample size was relatively small, and a multicenter experiment with a large sample size is needed to confirm our conclusions. Second, we only evaluated the parameters after 2 courses, and more time points should be chosen to obtain the best time points for evaluating the treatment efficacy. Third, it may not be objective enough using treatment-response criteria to evaluate the prognostic ability. We will attempt to explore the potential relationship between DWI, DKI derived parameters and OS in the following study. Finally, we only summarized patients treated with two first-line chemotherapy regimens, which could have led to selection bias, and more treatment schemes should be included in future evaluations.

### Summary

In conclusion, DWI and DKI parameters showed good diagnostic performance in differentiating PR and non-PR groups in patients with unresectable pancreatic cancer. Compared with DWI parameters, the MD_change_ among DKI parameters showed greater specificity in evaluating treatment response. Combined model SUM_change_ performed better than singe DWI and DKI model. DKI parameters could become new indicators for clinical efficacy evaluation while these results require further validation with a larger cohort.

## Data Availability

The datasets used and/or analysed during the current study are available from the corresponding author on reasonable request.

## Abbreviations

DKI: Diffusion kurtosis imaging
DWI: Diffusion weighted imaging
ADC: Apparent diffusion coefficient
PDAC: Pancreatic ductal adenocarcinoma
ROI: Region of interest
ROC: Receiver operator characteristic
AUC: Area under curve
MD: Mean diffusion
RECIST: Response evaluation criteria in solid tumours
MK: Mean kurtosis
PR: Partial response
CR: Complete response
SD: Stable disease
PD: Progressive disease
